# Targeting the Redox Balance Pathway Using Ascorbic Acid in *sdhb* Zebrafish Mutant Larvae

**DOI:** 10.3390/cancers13205124

**Published:** 2021-10-13

**Authors:** Margo Dona, Maaike Lamers, Svenja Rohde, Marnix Gorissen, Henri J. L. M. Timmers

**Affiliations:** 1 Department of Internal Medicine, Radboud University Medical Center, 6525 GA Nijmegen, The Netherlands; mhg.lamers@gmail.com (M.L.); srohde.99@gmail.com (S.R.); henri.timmers@radboudumc.nl (H.J.L.M.T.); 2 Department of Animal Ecology and Physiology, Radboud Institute for Biological and Environmental Sciences, Radboud University, 6525 AJ Nijmegen, The Netherlands; M.Gorissen@science.ru.nl

**Keywords:** phaeochromocytoma, paraganglioma, cancer, mitochondrial complex II, zebrafish, therapy, drug discovery, redox balance pathway, Vitamin C

## Abstract

**Simple Summary:**

Thus far, no curative therapies are available for malignant *SDHB*-associated phaeochromocytomas and paragangliomas (PPGLs). Therapy development is severely hampered by the limited availability of suitable animal models. In this study, we investigated the potential of the *sdhb^rmc200^* zebrafish model to study *SDHB*-associated PPGLs using a drug screening approach. One of the key features of cancer initiation and progression is redox imbalance. First, we identified increased reactive oxygen species levels in homozygous *sdhb^rmc200^* larvae at baseline. Next, we tested the effect of anti- and pro-oxidant ascorbic acid (Vitamin C) on these larvae. We validated the *sdhb^rmc200^* zebrafish model as a powerful drug screening tool to provide valuable insights into pathomechanisms, which may lead to novel therapeutic targets and therapy development in the future.

**Abstract:**

Patients with mutations in the β-subunit of the succinate dehydrogenase (*SDHB*) have the highest risk to develop incurable malignant phaeochromocytomas and paragangliomas (PPGLs). Therapy development is hindered by limited possibilities to test new therapeutic strategies in vivo. One possible molecular mechanism of *SDHB*-associated tumorigenesis originates in an overproduction of reactive oxygen species (ROS) due to mitochondrial dysfunction. Ascorbic acid (Vitamin C) has already been shown to act as anti-cancer agent in several clinical trials for various types of cancer. In this study, the potential of the *sdhb^rmc200^* zebrafish model to study *SDHB*-associated PPGLs using a drug screening approach was investigated. First, we identified increased basal ROS levels in homozygous *sdhb* larvae compared to heterozygous and wild-type siblings. Using a semi high-throughput drug screening, the effectiveness of different dosages of anti- and pro-oxidant Vitamin C were assessed to evaluate differences in survival, ROS levels, and locomotor activity. Low-dosage levels of Vitamin C induced a decrease of ROS levels but no significant effects on lifespan. In contrast, high-dosage levels of Vitamin C shortened the lifespan of the homozygous *sdhb^rmc200^* larvae while not affecting the lifespan of heterozygous and wild-type siblings. These results validated the *sdhb^rmc200^* zebrafish model as a powerful drug screening tool that may be used to identify novel therapeutic targets for *SDHB*-associated PPGLs.

## 1. Introduction

The mitochondrial enzymatic succinate dehydrogenase (SDH) complex, also called mitochondrial complex II, has an essential role in ATP production. The dysfunction of the SDH complex is linked to several diseases, varying from severe neuromuscular disorders [1] to different types of cancer including phaeochromocytomas and paragangliomas (PPGLs), gastrointestinal stromal tumour, renal cell carcinoma (RCC), pituitary adenoma, and pancreatic neuroendocrine tumours [2,3].

PPGLs are rare neuroendocrine tumours originating from chromaffin cells in the adrenal medulla or from extra-adrenal paraganglia, respectively [4]. The incidence of PPGLs is up to eight per million persons per year [5]. Although the majority of the tumours are benign, genetic predisposition can be a risk factor for metastasis development, resulting in poor prognosis [6,7,8,9]. The most prevalent *succinate dehydrogenase subunit B* (*SDHB*) germline mutations are especially known to play a crucial role in the pathogenesis of aggressive PPGLs, with a metastatic rate of 50–97% [9,10,11]. In general, the curative surgical removal of the tumour is no longer valid when metastases develop. Although not curative, chemotherapy, radionuclide therapy, and anti-angiogenic drugs might lead to the stabilisation of the disease for months to years, improved quality of life, and prolonged survival. To develop more effective and targeted treatment detailed insight into the pathomechanisms is essential [12].

Several hypotheses of the predisposition for the malignancy of *SDHB*-mutated PPGLs have been proposed [13,14]. Upon the dysregulation of the SDH complex, the oncometabolite succinate accumulates, which leads to the reprogramming of cellular metabolic pathways including hypermethylation, the activation of the HIF pathway, and decreased DNA repair [14]. In addition, the substantial loss of complex II activity impairs electron transfer to oxygen and thus leads to the increased formation of reactive oxygen species (ROS) and redox imbalance [9,15,16,17,18,19]. Increased ROS levels can cause defects in cell signalling, DNA damage, and lipid peroxidation [20]. The ability of ROS to cause genomic instability is a well-established cause of carcinogenesis. In this study, we investigated the potential of the *sdhb^rmc200^* zebrafish model to study *SDHB*-associated PPGLs using a drug screen approach.

High-dosage levels of ascorbic acid (Vitamin C) have already been shown to act as anti-cancer agent for several types of cancer [21]. Vitamin C can act as an antioxidant, reducing ROS levels, but it can also function as pro-oxidant to kill cancer cells in vitro and slow tumour growth in vivo. Pharmacologic levels of Vitamin C have been shown to aggravate the ROS-mediated toxicity in *SDHB^KD^* mouse phaeochromocytoma (MPC) cells, thus leading to genetic instability and apoptotic cell death [19]. Moreover, these *SDHB^KD^* MPC cells were injected into athymic nude mice, establishing metastatic PPGL tumours in vivo; the supplementation of high-dosage levels of Vitamin C strongly delayed metastatic lesions and thereby improved disease outcome [19].

Recently, we generated and characterised a systemic *sdhb^rmc200^* knockout zebrafish model that mimics the metabolic properties of *SDHB*-associated PPGLs [22]. Homozygous *sdhb^rmc200^* mutant larvae display a decreased lifespan due to decreased mitochondrial complex ΙΙ activity and significant succinate accumulation, and they mimic important genomic and metabolic effects observed in *SDHB*-associated PPGL tumours [22]. In addition, a decreased mobility attributed to energy deficiency is observed. These phenotypic read-outs in 6-day-old zebrafish larvae can be used to evaluate the effects of candidate drugs and could facilitate the (semi) high-throughput in vivo testing of potential therapeutic agents for *SDHB*-associated PPGLs.

In this study, we investigated redox homeostasis in larvae of the *sdhb^rmc200^* zebrafish model, and we evaluated the effect of both low-dosage and high-dosage levels of Vitamin C by using an in vivo zebrafish drug screen.

## 2. Results

### 2.1. sdhb^rmc200^ Zebrafish Larvae as Drug Screening Model for SDHB-Associated PPGLs

#### 2.1.1. Homozygous *sdhb^rmc200^* Zebrafish Larvae Exhibit Increased Reactive Oxygen Species (ROS) Levels

**Figure 1:**
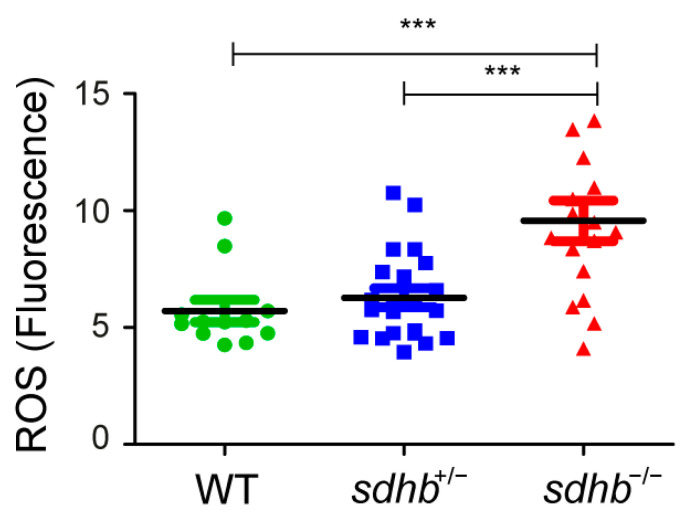
Reactive oxygen species (ROS) measurements showed a significant increase in homozygous *sdhb* larvae (*n* = 17) compared to their heterozygous (*n* = 22) and wild-type siblings (*n* = 12) at 6 dpf. One-way ANOVA with Tukey’s post hoc test, *** *p* < 0.001.

To investigate whether *sdhb^rmc200^* larval zebrafish mutants possess an unbalanced cellular redox state, whole-mount ROS-detection was used to determine ROS levels at baseline. At day 6 post fertilization (dpf), increased levels of ROS were observed in homozygous *sdhb* compared to their heterozygous *sdhb* and wild-type siblings (Figure 1).

#### 2.1.2. Successful Design of Drug Screening Protocol

**Figure 2:**
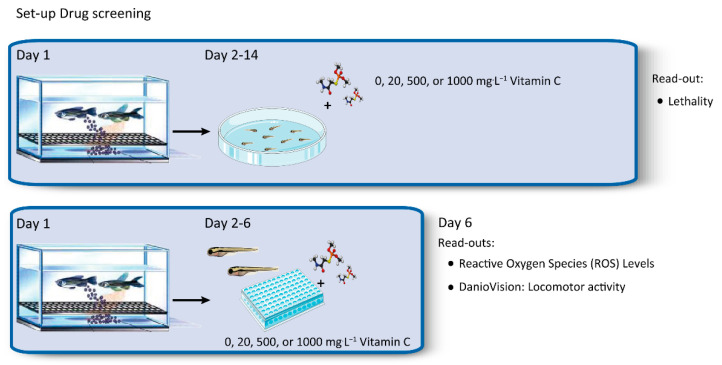
Schematic overview of zebrafish drug screening set-up.

To test the effect of Vitamin C on zebrafish larvae, an optimal drug screening protocol was established (Figure 2). First, the offspring of the incross of heterozygous adult *sdhb* mutants were collected. At day 2 post fertilization (dpf), hatched larvae were assembled in 48-well plates with either an E3 control egg medium or E3 medium supplemented with Vitamin C (20, 500, or 1000 mg⋅L^−1^). Different read-outs to assess the effect of the vitamin were developed. Lethality scores were performed to assess the effect of Vitamin C on overall survival. In addition, at day 6, ROS levels were measured to check the effect of supplementation of the anti- and pro-oxidant Vitamin C. As quick read-out for general health and toxicity, locomotor activity was evaluated using DanioVision at day 6.

### 2.2. Effects of Vitamin C as Anti- and Pro-Oxidant on Sdhb Zebrafish Larvae

#### 2.2.1. High-Dosage Levels of Vitamin C Decreases Lifespan of Homozygous *sdhb^rmc200^* Larvae

**Figure 3:**
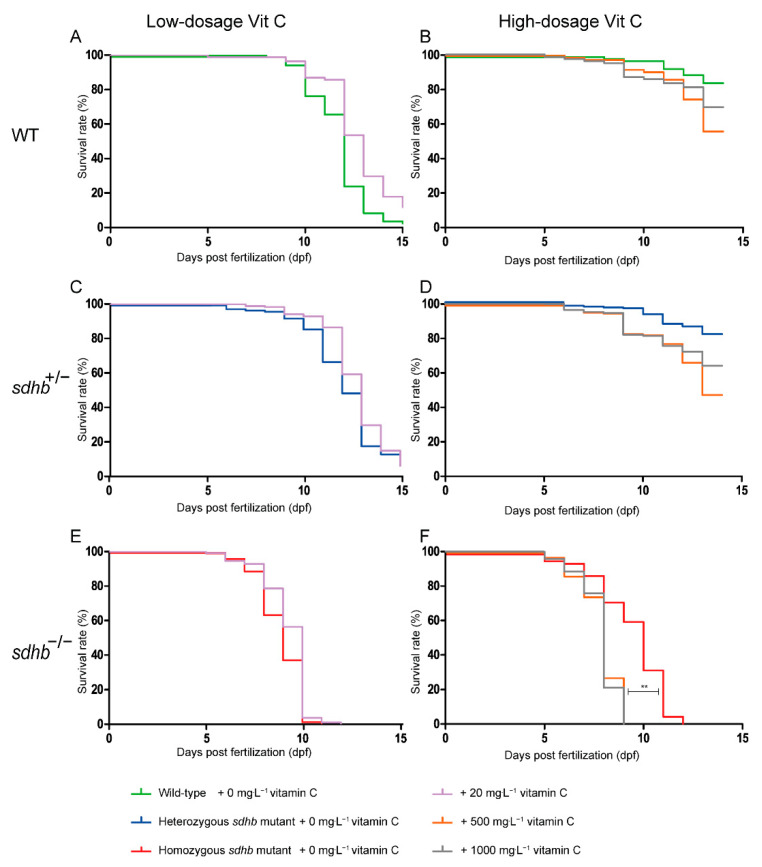
Survival in WT siblings (**A**,**B**), heterozygous *sdhb* mutants (**C**,**D**), and homozygous *sdhb* mutants (**E**,**F**) presented for control (0 mg⋅L^−1^ green, blue, and red lines, respectively); low dosage ((**A**,**C**,**E**) 20 mg⋅L^−1^, purple line) and high dosage ((**B**,**D**,**F**) 500 mg⋅L^−1^, orange line; 1000 mg⋅L^−1^, grey line) Vitamin-C-treated larvae. (**A**,**C**,**E**) Survival rate (%) was not significantly prolonged after the low-dosage treatment of Vitamin C *sdhb* larvae compared to control (E3 medium supplemented; 0 mg⋅L^−1^) larvae (control: *n* = 95 homozygous *sdhb* larvae, *n* = 126 heterozygous *sdhb* larvae, and *n* = 85 WT larvae; 20 mg⋅L^−1^ Vitamin C: *n* = 112 homozygous *sdhb* larvae, *n* = 190 heterozygous *sdhb* larvae, and *n* = 91 WT larvae from two replicates). (**B**,**D**,**F**) Survival rate (%) was significantly reduced in homozygous *sdhb* mutants after treatment with high dosages of Vitamin C (500 and 1000 mg⋅L^−1^) compared to control (E3 medium supplemented) larvae (control: *n* = 72 homozygous *sdhb* larvae, *n* = 198 heterozygous *sdhb* larvae, and *n* = 87 WT larvae; 500 mg⋅L^−1^ Vitamin C: *n* = 84 homozygous *sdhb* larvae, *n* = 195 heterozygous *sdhb* larvae, and *n* = 70 WT larvae; 1000 mg⋅L^−1^ Vitamin C: *n* = 90 homozygous *sdhb* larvae, *n* = 172 heterozygous *sdhb* larvae, and *n* = 86 WT larvae from two replicates). No significant differences were observed between *sdhb* heterozygous and WT siblings at either low or high dosage Vitamin C treatment. Log-rank (Mantel–Cox) test, ** *p* < 0.01.

Heterozygous adult *sdhb* mutants were used to generate a mixed offspring following Mendelian inheritance. The larvae were checked at least twice a day to determine lethality. At baseline, homozygous *sdhb* larvae showed an enhanced mortality compared to heterozygous and wild-type siblings (Figure 3A,C,E). Low dosages of Vitamin C (20 mg⋅L^−1^) did not have an significant effect on the survival of homozygous *sdhb*, heterozygous *sdhb*, or wild-type larvae measured until two weeks of age. High dosage levels of Vitamin C (500 and 1000 mg⋅L^−1^) significantly decreased the survival rate of the homozygous *sdhb* mutants (Figure 3F; *p* < 0.01) while not having a significant effect on heterozygous and wild-type siblings (Figure 3B–D).

#### 2.2.2. Effects of Vitamin C Treatment on ROS Levels

**Figure 4:**
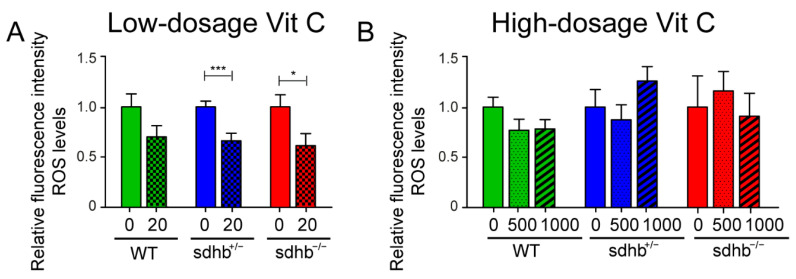
Reactive oxygen species (ROS) measurements after a low- and high-dosage Vitamin C treatment. Relative average fluorescence levels are shown normalised to control levels without Vitamin C supplementation. (**A**) Larvae were supplemented with 20 mg⋅L^−1^ Vitamin C homozygous *sdhb* (*n* = 22), heterozygous sibling (*n* = 33), and wild-type sibling (*n* = 21) compared to the control group (indicated with 0 mg⋅L^−1^) consisting of homozygous *sdhb* larvae (*n* = 24), heterozygous sibling (*n* = 38), and wild-type sibling (*n* = 18) from two different replicates measured at 6 dpf. Low-dosage levels of Vitamin C significantly decreased ROS levels in heterozygous and homozygous *sdhb* larvae compared to the untreated control group. (**B**) Larvae were supplemented with 500 mg⋅L^−1^ Vitamin C homozygous *sdhb* (*n* = 12), heterozygous sibling (*n* = 24), and wild-type sibling (*n* = 18); 1000 mg⋅L^−1^ Vitamin C homozygous *sdhb* (*n* = 8), heterozygous sibling (*n* = 22), and wild-type sibling (*n* = 22) compared to the control group (indicated with 0 mg⋅L^−1^) consisting of homozygous *sdhb* larvae (*n* = 10), heterozygous sibling (*n* = 14), and wild-type sibling (*n* = 16) from three different replicates measured at 6 dpf. High-dosage levels of Vitamin C did not alter ROS levels in all three genotypes compared to the untreated control group. Two-tailed unpaired Student’s *t*-test, * *p* < 0.05 and *** *p* < 0.001.

The effect of low-dosage and high-dosage levels of Vitamin C was measured compared to an untreated control group. In Figure 4, the ROS levels after the supplementation of either the E3 medium control medium (0 mg⋅L^−1^) or low-dosage levels of Vitamin C (20 mg⋅L^−1^) and high-dosage Vitamin C levels of Vitamin C (500 and 1000 mg⋅L^−1^) are shown. Low-dosage Vitamin C levels significantly decreased ROS levels in the heterozygous and homozygous *sdhb* mutant group compared to the untreated control group, while no significant differences were identified in the wild-type group without low-dosage levels of Vitamin C. High-dosage levels of Vitamin C possessed a more heterozygous effect in heterozygous and homozygous *sdhb* larvae. No significant differences were identified high-dosage levels of Vitamin C supplementation.

#### 2.2.3. Behavioural Assessment as Quick Read-Out of Toxicity of the Larvae

**Figure 5:**
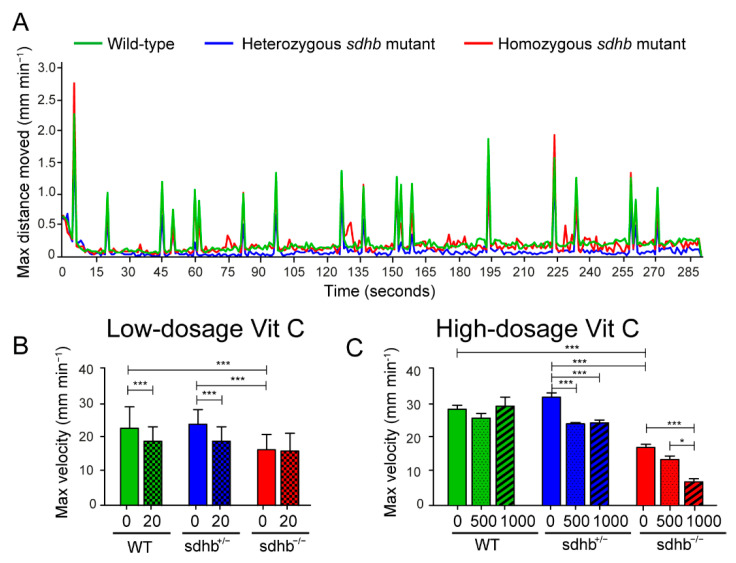
Startle response measurements at basal level and after low- and high-dosage levels of Vitamin C. (**A**) Optimized short protocol (<5 min in total) to quickly assess startle responses as a toxicity indicator induced by tapping stimuli with random intervals varying between 2 and 35 s. The max distance moved (mm min^−1^) is plotted against time per seconds. Between the wild-type (green line) and heterozygous mutants (blue line), no differences were observed, while homozygous *sdhb* mutants (red line) showed a decrease in moved distance. (**B**) Quantification of the average of the maximum velocity of three startle responses with or without the supplementation of low-dosage levels of Vitamin C (20 mg⋅L^−1^). Low-dosage levels of Vitamin C decreased the startle response of wild-type and heterozygous *sdhb* larvae while not affecting homozygous *sdhb* larvae. Larvae were supplemented with 20 mg⋅L^−1^ Vitamin C homozygous *sdhb* (*n* = 106), heterozygous sibling (*n* = 244), and wild-type sibling (*n* = 137) compared to the control group (indicated with 0 mg⋅L^−1^) consisting of homozygous *sdhb* larvae (*n* = 91), heterozygous sibling (*n* = 227), and wild-type sibling (*n* = 100) from five different replicates measured at 6 dpf. (**C**) Quantification of the average of the maximum velocity of three startle responses with or without supplementation of high-dosage levels of Vitamin C (500 and 1000 mg⋅L^−1^). Both 500 and 1000 mg⋅L^−1^ concentrations of Vitamin C induced a decreased startle response in heterozygous *sdhb* larvae, while only the 1000 mg⋅L^−1^ concentration of Vitamin C induced a decreased startle response in homozygous *sdhb* larvae and none of the high-dosage levels of Vitamin C significantly altered the startle response of the wild-type larvae. Larvae were supplemented with 500 mg⋅L^−1^ Vitamin C homozygous *sdhb* (*n* = 73), heterozygous sibling (*n* = 139), and wild-type sibling (*n* = 55), 1000 mg⋅L^−1^ Vitamin C homozygous *sdhb* (*n* = 20), heterozygous sibling (*n* = 37), and wild-type sibling (*n* = 19) compared to the control group (indicated with 0 mg⋅L^−1^) consisting of homozygous *sdhb* larvae (*n* = 32), heterozygous sibling (*n* = 48), and wild-type sibling (*n* = 40) from at least two different replicates measured at 6 dpf. One-way ANOVA with Tukey’s post hoc test, * *p* < 0.05 and *** *p* < 0.001.

We previously identified that homozygous *sdhb* mutant larvae possess a lower basal activity and reduced endurance compared to heterozygous and wild-type siblings [22]. We designed a short protocol (<5 min) with randomized tapping stimuli to induce a robust startle response. In Figure 5A, the maximum distance moved is plotted against time in seconds, all peaks reflect a startle response induced by the tapping stimulus. At baseline, homozygous *sdhb* mutant larvae possessed a decreased startle response compared to heterozygous and wild-type siblings. Low-dosage levels of Vitamin C induced a significantly decreased startle response in heterozygous *sdhb* and wild-type larvae but did not alter the startle response of homozygous *sdhb* larvae (Figure 5B). Furthermore, both high-dosage levels of Vitamin C decreased the startle response of heterozygous *sdhb* larvae, but only 1000 mg⋅L^−1^ had a significant decreased in startle response in homozygous *sdhb* larvae and none of the high-dosage levels significantly affected the startle response of the wild-type larvae (Figure 5C).

## 3. Discussion

The development of novel therapeutic targets for metastatic *SDHB*-associated PPGLs is hampered by the limited availability of suitable in vivo models. In this study, we investigated the effects of Vitamin C in the *sdhb^rmc200^* zebrafish model and thereby tested the suitability for drug screening experiments in this validated PPGL cancer model [22]. First, we revealed an imbalance in cellular redox homeostasis in homozygous *sdhb* zebrafish larvae by increased levels of ROS at baseline. Next, we successfully tested the effect of different dosages of Vitamin C treatment: aqueous solutions of Vitamin C were added to the swim water of the larvae, and we showed that although low-dosage levels (20 mg⋅L^−1^) of Vitamin C did not increase the lifespan of homozygous *sdhb* larvae, ROS was decreased. High-dosage levels of Vitamin C (500 and 1000 mg⋅L^−1^) significantly shortened the lifespan of homozygous *sdhb* larvae while not altering the lifespan of their heterozygous *sdhb* and wild-type siblings regardless of unchanged levels of ROS.

As previously reported, homozygous *sdhb* larvae have a shortened lifespan (maximal two weeks of age) compared to heterozygous *sdhb* and wild-type siblings [22]. Additionally, these homozygous *sdhb* larvae display key metabolic characteristics of *SDHB*-associated PPGLs such as impaired mitochondrial complex II function and vastly increased succinate levels [22]. The heterozygous *sdhb* larvae revealed no differences in mitochondrial function and metabolite levels compared to wild-types siblings.

Here, we identified increased ROS levels in homozygous *sdhb* larvae compared to heterozygous and wild-type siblings. Redox imbalance by increased levels of ROS is known to play a critical role in carcinogenesis [23,24,25], as has also been suggested for PPGLs [14,26,27]. Although no alternative relevant systemic *Sdhb* knockout animal model is available, different cell lines and graft models have been created. Our findings are in line with increased ROS levels in the mitochondria of *SDHB*-deficient mouse phaeochromocytoma cells [19], confirmed by two *SDHB*-silenced cell lines and one *SDHC*-mutated transgenic mouse cell line [17,28,29]. On the other hand, two other studies reported no increased ROS levels in cell lines silenced for *SDHB* [30,31], despite hypoxia-inducible factor (HIF) stabilisation. The usage of different cell lines and the variations of different assays for measuring ROS could be reasons for this discrepancy.

Zebrafish models possesses unique advantages for investigating the effect of drugs to unravel pathomechanisms and test the therapeutic efficacy of re-purposing drugs from related types of cancer such as neuroblastoma and RCC [32]. Zebrafish can produce a large number of offspring, rapidly develop, and still have a high grade of similarity with humans; approximately 70% of human genes have at least one obvious zebrafish orthologue [33]. The use of larval zebrafish as a model organism in semi high-throughput drug screens is rapidly expanding [34,35,36]. This drug screen approach enables one to test a high number of potential targets, evaluate toxicity, and evaluate compound efficiency to select the most promising drugs to be validated in pre-clinical tumour models. The read-outs we optimized for our drug screen are lethality measurements, which are the most important and direct values used to check effects on lifespan, a protocol to assess locomotion activity as read-out for toxicity and possible other negative side-effects, and ROS levels.

Vitamin C is a natural compound with a high safety profile that was previously positively tested in pre-clinical studies for non-PPGL types of cancer [37]. The efficiency of Vitamin C has also been assessed in clinical trials, such as renal cell carcinoma in a phase-II clinical trial [21]. Often, Vitamin C is used supplementary to other types of treatment such as chemotherapy and radiation therapy. The exact mechanism of its action remains unclear since multiple critical pathways are targeted including redox imbalance, epigenetic reprogramming, and oxygen-sensing regulation, thereby preventing ROS-mediated toxicity [21]. Pharmacological levels of Vitamin C aggravated the oxidative burden of *SDHB*-deficient PPGLs, leading to genetic instability and apoptotic cell death [19]. Furthermore, in a pre-clinical animal model with PPGL allografts, high-dosage levels of Vitamin C suppressed metastatic lesions and prolonged overall subject survival [19].

We investigated the effects of low- and high-dosage levels of Vitamin C as pro- and antioxidants in the *sdhb* zebrafish larvae. Low-dosage levels of Vitamin C induced a decrease of ROS levels in homozygous mutants but no significant effects on lifespan. In contrast, high-dosage levels of Vitamin C further shortened the lifespan of the homozygous *sdhb* larvae while not affecting heterozygous and wild-type siblings. This is in line with previous findings obtained in the allografted mice model treated, with high-dosage levels of Vitamin C inducing ROS-medicated toxicity in tumour cells [19]. We detected no increase in basal ROS levels in the homozygous *sdhb* larvae supplemented with high-dosage levels of Vitamin C at 6 dpf. This discrepancy could be explained by the timing of the ROS measurement. We performed ROS measurements by using the fluorescent dye CM-H2DCFDA analysed by microscopy at 6 dpf. Fluorescence-activated cell sorting (FACs) analysis could optimise the quantification of the total ROS value in an entire larvae, and the measurements also could be performed at later time points to identify possible differences. Since in all other studies, HIF stabilisation was detected regardless of whether increased or normal ROS levels were detected, in follow up studies, other ROS indicators and HIF stabilisation could be measured as well.

Despite the fact that homozygous *sdhb* zebrafish larvae mimic the metabolic human tumour environment, the major limitation of using this zebrafish model is the absence of tumours at the age of 14 days. The translational value to predict the effect in human PPGLs therefore remains challenging and requires further investigation. For this, the allograft mice model and rat xenograft model are currently the best used alternatives [19,38]. Currently, the homozygous *sdhb* zebrafish larvae possess resemblance to patients with bi-allelic *SDHB* mutations with a Leigh-syndrome-like phenotype, resulting in severe progressive neurodegeneration and myopathy with the onset in infancy and poor prognosis [1,39]. The supplementation of low-dosage levels of Vitamin C was also shown to be effective in pre-clinical studies for neuropathy [40], and other anti-oxidants showed a beneficial effect for patients with mitochondrial disorders [41]. More research is required to investigate the therapeutic potential of low-dosage levels of Vitamin C for Leigh-syndrome-like patients.

To follow up the PPGL research, we will investigate whether adult heterozygous *sdhb* fish develop tumours in comparison to human *SDHB* mutations, which are at risk of developing PPGLs. If successful, this zebrafish tumour model can be complementarily used to test the potential of the most promising compounds identified in the larval drug seen for the effectiveness on tumour growth and to further unravel the mode of action behind its pathomechanism. Additionally, the onset of tumorigenesis and the prevention of tumour formation could be investigated in more detail.

In this study, we identified increased ROS levels in our homozygous *sdhb* larvae at baseline. Further, we validated the zebrafish larvae drug screen as tool to screen for therapeutic compounds and possible combination of compounds to target pathways involved in the tumorigenesis of *SDHB*-associated PPGLs. The most powerful advantage of this zebrafish model is its ability to screen many targets for possible new therapeutics in a cheap and cost-effective manner. This enables us to narrow down possible therapeutics to test for effectiveness in a more advanced tumour model. Patient-derived xenografts (PDXs) or cancer “Avatars” could attribute to personalized medicine in the future. In addition to mouse and rat PDXs [38,42], zebrafish Avatars are emerging as a cheaper and faster alternative [43,44] to hopefully accelerate personalised drug discovery for currently incurable metastatic *SDHB*-associated PPGLs.

## 4. Materials and Methods

### 4.1. Zebrafish Maintenance and Husbandry

Experimental procedures were conducted in accordance with institutional guidelines and National and European laws. Ethical approval of the experiments was granted by Radboud University’s Institutional Animal Care and Use Committee (IACUC, application numbers RU-DEC 2015-0098 and RU-DEC 2020-0030). Wild-type adult Oregon AB* zebrafish (*Danio Rerio*) and heterozygous adult *sdhb^rmc200^* mutants were used [22]. Eggs were obtained from natural spawning. Larvae were maintained and raised by standard methods [45].

### 4.2. Genotyping

Larvae were briefly anesthetised in 2-phenoxyethanol (0.1%, *v/v*). Genomic DNA isolation and PCR amplification and analysis were performed as previously described [22].

### 4.3. ROS Measurements

ROS levels were assessed in 6 dpf zebrafish larvae using the 2′,7′-dichlorodihydrofluorescein diacetate (CM-H2DCFDA) dye (Fisher Scientific). When oxidized, this non-fluorescent dye is converted into a fluorescent compound, 2′,7′-dichlorofluorescein (DCF) [33]. The ROS levels were measured according to protocol [33]. In brief, each larva was individually placed in a well of a 96-well plate with 100 μL of an E3 embryo medium at 6 dpf. A working solution of H2DCFDA (500 μg/mL in dimethyl sulfoxide (DMSO, 14.1 M)/E3 medium (5 mM NaCl, 0.17 mM KCl, 0.33 mM CaCl2, and 0.33 mM MgSO4)) was prepared, and 100 μL were added to each well. Then, the solutions were mixed for 20 s at 150 rpm and incubated for 3.5 h at 28 °C in the dark. After incubation, the plates were analysed with the use of a fluorescence microscope (EVOS M5000 Imaging System) for the low-dosage levels of Vitamin C and a fluorescence microscope (Leica MZFL-III) for the high-dosage levels of Vitamin C. The level of fluorescence was calculated with the use of ImageJ [34].

### 4.4. Vitamin C Treatments

Fertilised eggs originating from a heterozygous *sdhb^rmc200^* incross were reared in petri dishes filled with E3 medium supplemented with 0.1% methylene blue (Sigma-Aldrich) and incubated at 28 °C with a day/night rhythm. At 2 dpf, the hatched larvae were put in a 48-wells plate containing 200 μL of medium with or without Vitamin C (A4544, Sigma-Aldrich) until 6 dpf. At day 5, the medium was replaced with E3 medium without or with appropriate concentrations of Vitamin C. All working solutions (20, 500, or 1000 mg⋅L^−1^) were freshly prepared in E3 medium, and the pH was adjusted using 0.5 M NaOH between 6.8 and 8.5 [46].

### 4.5. Lethality Score Analysis

Heterozygous *sdhb^rmc200^* adult fish were crossed to collect eggs. The larvae were divided into two groups. An E3 medium was added for the control group, and a Vitamin C dosage (20, 500, or 1000 mg⋅L^−1^) was added from 2 dpf onwards. Larvae were either raised in petri dishes (max 60 larvae per dish) for low-dosage levels of Vitamin C experiments or transferred to 1 L tanks for high-dosage levels of Vitamin C experiments. Minimally, twice a day, the larvae were checked to collect death larvae. Death larvae were collect in 75 µL of lysis buffer (40 mM NaOH and 0.2 mM EDTA) and then genotyped. Every day, the medium was refreshed, and in the afternoon, the larvae were fed with Gemma micro 75 ZF for the low-dosage level Vitamin C experiments and with rotifers for the high-dosage level Vitamin C experiments.

### 4.6. Behavioural Assessment: Locomotion Assay

Using DanioVision (Noldus Information Technologies, Wageningen, The Netherlands), the locomotion of 6 dpf larvae was tracked. Each larva was individually placed in a well of a 48-well plate with 200 μL of an E3 embryo medium. The study was conducted at a constant 28 °C and 3000 lux. The short protocol (<5 min in total) to induce startle responses consisted of tapping stimuli with random intervals varying between 2 and 35 s. Afterward, the complete larval body was used for genotyping. Larvae were pooled based on genotype and data grouped per phenotype were exported to Microsoft Excel (version 1906). The max velocity was used as read-outs for startle response to detect possible differences between the three different genotypes and Vitamin C treatment.

### 4.7. Statistical Analysis

GraphPad Prism software (Version 5.03 for Windows, GraphPad Software, La Jolla, CA, USA) was used to generate scatter plots, calculate mean values, and perform statistical analyses. The Log-rank (Mantel–Cox) test was used for the survival curve analysis. The one-way ANOVA with Tukey’s post hoc test was used for the ROS basal levels and startle response quantification. A two-tailed unpaired Student’s *t*-test was used for ROS levels without or with Vitamin C treatment.

## 5. Conclusions

In this study, we showed that the *sdhb* zebrafish model possesses an unbalanced redox homeostasis, as indicated by elevated ROS levels at baseline. Further, we evaluated the utility of *sdhb* zebrafish larvae to test drugs for their therapeutic potential for *SDHB*-associated PPGLs. We demonstrated that high-dosage levels of Vitamin C shortened the lifespan of homozygous *sdhb* larvae. This zebrafish model could potentially be used for preclinical drug screening and the identification of new therapeutic targets.

## Data Availability

The data presented in this study are available on request from the corresponding author.
